# All cholesterol-lowering interventions are expected to reduce stroke: Confirmatory data from IMPROVE-IT

**DOI:** 10.1016/j.dib.2016.04.059

**Published:** 2016-04-27

**Authors:** Raffaele De Caterina, Tanya Salvatore, Roberto Marchioli

**Affiliations:** a Institute of Cardiology and Center of Excellence on Aging, “G. d׳Annunzio” University, Chieti, Italy; b Hematology and Oncology, Therapeutic Science and Strategy Unit, Quintiles, Milan, Italy

**Keywords:** Epidemiology, Cholesterol, Cholesterol lowering, Statins, Stroke, Risk prediction, Ezetimibe

## Abstract

The relationship of cholesterol with stroke is much less clear than its relationship with myocardial infarction, thus confounding the interpretation of results with cholesterol-lowering trials (Di Napoli et al., 2002) [1], (De Caterina et al., 2010) [2]). IMPROVE-IT data ((Cannon et al. 2015) [3]), showing a 13.3% reduction in total cholesterol at one year in association with a hazard ratio (HR) of 0.i86 for total stroke during the trial, are very closely aligned with the relative risk of 0.90 predicted based on the totality of lipid lowering interventions ((De Caterina et al., 2016) [4]). We here provide the data from the original trials used to construct this meta-analysis, with the now added additional data from IMPROVE-IT, well-fitting the previously found meta-regression line.

These data are important to predict stroke outcomes in currently ongoing trials now testing PCSK9 or cholesterol ester transfer protein inhibitors.

**Specifications Table**

**Table:** 

Subject area	*Biology/Medicine*
More specific subject area	*Lipid-lowering intervention trials in cardiovascular disease prevention*
Type of data	*Table*
How data was acquired	*Literature data extraction*
Data format	*Raw*
Experimental factors	*No data pretreatment-calculation of percent reduction in stroke as a function of percent reduction in total cholesterol in each of the original source trials*
Experimental features	*Meta-regression now fitting the recently published IMPROVE-IT data into the meta-regression*
Data source location	*n/a*
Data accessibility	*Data are within this article*

**Value of the data**

• These data, as obtained through already published literature reinforce the idea that also reduction of stroke, contrary to what previously believed, can be explained by reduction in cholesterol (total cholesterol in this analysis), both in statin and non-statin trials.
• Recently published data from IMPROVE-IT perfectly fit the regression line from other source data on such relationship.
• These results now allow a prediction of the reduction in stroke in trials now testing the PCSK9 inhibitors and the cholesterol ester transfer protein (CETP) inhibitor anacetrapib.

## 1 Data

Data provided here are the characteristics of source trials used for the construction of the meta-regression of reduction in stroke as a function of reduction in total cholesterol.

## 2 Experimental design, materials and methods

We had previously carried out a meta-regression by using inverse variance weighted linear regression of the log RR for total stroke against the percent of TC reduction as the explanatory variable. Weights in each study were the reciprocals of the variances for the logarithm RR for stroke. The meta-regression had yielded the following equation:

ln(totalstrokeRR)=0.00518−0.00793(%TCreduction)

The regression coefficient for percent TC reduction was significantly different from zero (*p*=0.0017). This equation indicates that some benefit from cholesterol-lowering intervention on the risk of stroke can be expected when the percent reduction of serum cholesterol is >2–3%, the clinical benefit becoming statically significant when TC is reduced by ~8%.

We have now compared the reduction in stroke observed in IMPROVE-IT [3] with that calculated from our previous meta-analysis of all lipid lowering interventions reporting effects on stroke, in all trials as previously reported and as shown in Table 1. IMPROVE-IT [3] has now shown that a 13.3% reduction in total cholesterol at one year was associated with a hazard ratio (HR) of 0.86 for total stroke during the trial. This result is very closely aligned with the relative risk of 0.90 predicted on the basis of the totality of lipid lowering interventions [2], [5].

## 3 Original Source data

.

## Figures and Tables

**Table 1:** Description of the trials selected – demographic characteristics.

**TRIAL, [ref No], Year of publication**	**Design**^a^	**Follow-up**^b^	**Total patients**	**Total stroke**	**Fatal stroke**	**Non-fatal stroke**	**AGE (mean)**	**SMK (%)**	**DM (%)**	**HBP (%)**	**PMI (%)**	**PST (%)**
Oslo [6], [7], [8],1966	D,op,SE	*5*	412	3	2	NA	56.0	64.6	10.0	–	100	–
MRC [9], [10],1968	D,op,SE	4	393	2	2	0	–	82.5	0.0	13.0	100	–
LA [11],1969	D,b,PS	*8*	846	38	12	26	65.5	66.4	–	–	20.1	12.5
Newcastle [12],1971	F,b,SE	3.6	497	1	1	0	52.5	65.0	0.0	0.0	23.0	–
Scottish [13], [14],1971	F,b,SE	3.4	717	5	5	0	52.1	56.6	0.0	–	72.9	–
VA [15],1974	F,b,SE	4.5	532	60	13	NA	–	–	23.5	64.5	–	16.0
CDP [16], [17], [18], [19],1975	F O,b,SE	6.2	5011	161	34	NA	52.0	37.9	5.0	20.0	100.0	2.0
Dorr [20],1978	O,b,PS	1.9	1094	1	1	0	50.5	–	13.7	16.2	6.2	0.5
WHO [21], [22], [23], [24],1980	F,b,PR	5.3	10627	NA	25	31	45.9	56.0	0.0	0.0	0.0	0.0
McCaughan [25],1981	O,b,PS	*1*	118	0	0	NA	49.8	44.6	–	–	33.9	–
LRC-CPPT [26],1984	O,b,PR	7.4	3806	35	4	NA	47.7	37.5	0.0	0.0	0.0	0.0
CLAS I [27], [28],1987	O,b,SE	*2*	188	0	0	0	54.2	0.0	0.0	0.0	–	–
Helsinki [29], [30],1987	F,b,PR	5	4081	10	10	0	47.3	36.2	2.6	14.0	0.0	–
Stockholm [31], [32],1988	O,op,SE	*5*	555	11	6	5	59.8	67.3	3.3	36.0	100.0	–
Minnesota [33],1989	D,b,PR	1.1	9057	43	43	NA	48.0	–	–	–	–	–
FATS [34],1990	S O,b,SE	2.7	98	0	0	NA	47.3	23.9	0.0	34.7	43.6	–
POSCH [35], [36], [37], [38],1990	B,op,SE	9.7	838	29	3	NA	51.0	35.0	0.1	0.0	100.0	0.0
EXCEL [39], [40], [41],1991	S,b,PS	*0.9*	8245	11	1	NA	55.8	18.3	1.1	39.6	–	3.9
Singh [42], [43],1992	D,b,SE	1	406	3	3	0	51.3	35.4	18.0	22.0	100.0	–
Frick [44],1993	F,b,SE	*5*	628	2	2	0	48.6	38.8	–	–	9.0	–
MARS [45], [46],1993	S,b,SE	2.2	270	3	0	NA	58.0	–	0.0	46.0	60.0	–
PMSG-CRP [47],1993	S,b,SE	*0.5*	1062	3	0	3	55.0	28.7	0.0	47.5	34.5	–
4 S [48], [49], [50],1994	S,b,SE	5.5	4444	132	26	NA	58.6	25.6	4.5	26.0	79.3	0.0
ACAPS [51],1994	S,b,PR	2.8	919	5	2	3	61.7	11.9	2.3	28.8	0.0	0.0
CCAIT [52],1994	S,b,SE	*2*	331	1	0	NA	53.0	27.0	14.0	37.0	54.0	18.0
LR [53],1994	S,b,SE	0.5	404	1	0	1	62.0	49.8	11.6	48.8	25.0	–
Lyon [54],1994	D,b,SE	2.3	605	3	0	3	53.5	6.2	–	0.0	100.0	–
MAAS [53],1994	S,b,SE	*4*	381	3	0	NA	55.3	23.9	0.0	–	54.3	–
PLAC-I [55], [56], [57], [58],1994	S,b,SE	2.3	408	2	0	2	57.0	16.5	0.0	45.5	43.5	0.0
PLAC-II [57], [59], [60],1994	S,b,SE	*3*	151	4	1	NA	62.5	12.1	–	0.0	63.8	–
Regress [61], [62],1994	S,b,SE	*2*	884	2	0	2	56.2	27.7	0.1	27.8	47.4	–
KAPS [63],1995	S,b,PS	*3*	447	6	1	5	57.4	26.2	2.5	33.1	7.6	–
CARE [64], [65],1996	S,b,SE	5	4159	128	16	NA	59.0	21.0	14.5	42.5	100	–
WOSCOPS [66],1996	S,b,PR	4.9	6595	97	10	NA	55.2	44.0	1.0	15.5	0.0	0.0
CIS [67],1997	S,b,SE	2.3	254	0	0	0	49.3	84.3	0.0	–	–	–
LOCAT [68],1997	F,b,SE	2.5	395	0	0	0	59.2	–	0.0	40.0	55.2	–
PCABGT [69],1997	S,op,SE	4.3	1351	34	NA	NA	61.5	11.3	8.6	–	49.3	–
PREDICT [70],1997	S,b,SE	*0.5*	695	1	1	0	58.3	33.7	7.2	30.7	37.1	1.9
AFCAPS [71],1998	S,b,PR	5.2	6605	31	NA	NA	58.0	12.4	2.4	21.9	0.0	0.0
LIPID [72], [73], [74],1998	S,b,SE	6.1	9014	373	49	NA	61.5	9.6	8.7	41.7	63.8	4.1
Mas [75],1999	O,b,SE	*0.5*	437	1	NA	NA	58.0	32.7	17.8	82.2	–	3.9
GISSI P [76],2000	S,op,SE	1.9	4271	39	8	31	60.0	11.9	13.6	36.5	100.0	–
SCAT [77],2000	S,b,SE	4.0	460	11	9	NA	61.0	15.0	10.9	35.2	70.4	–
VA-HIT [78],2000	F,b,SE	5.1	2531	134	12	NA	64.0	20.5	24.5	57.0	61.0	–
BCAPS [79],2001	S,b,SE	*3*	793	8	NA	NA	61.8	30.8	3.0	12.1	–	–
BIP [80],2001	F,b,SE	6.2	3090	149	NA	NA	60.1	11.8	10.0	32.4	77.9	1.1
DAIS [81], [82], [83],2001	F,b,SE	3.3	418	12	NA	NA	56.8	14.8	100.0	51.4	–	–
HATS [84],2001	S,b,SE	3	160	4	0	4	53.0	24.0	16.0	49.0	55	–
ALLHAT-LLT [85],2002	S,op,PR	4.8	10355	440	109	NA	66.4	23.2	35.1	100.0	0.0	–
FAST [86],2002	S O,PR	*2*	246	0	0	0	66.1	59.3	22.8	41.5	–	–
GREACE [87],2002	S,op,SE	3	1600	26	1	NA	58.5	–	19.6	42.9	81.2	–
HPS [88], [89],2002	S,b,SE	5	20536	1029	215	865	64.0	14.1	29.0	41.0	41.0	–
LEADER [90], [91],2002	F,b,SE	4.6	1568	109	22	97	68.2	37.8	17.1	–	19.8	11.7
LIEM [92],2002	S,b,SE	1	540	3	3	0	60.5	–	–	–	100.0	–
LIPS [93],2002	S,b,SE	3.9	1677	3	3	NA	60.0	26.6	12.1	38.6	44.4	2.6
PROSPER [94],2002	S,b,PS	2.2	5804	266	36	235	75.4	26.8	10.7	61.9	13.4	–
ALERT [95], [96],2003	S,b,PS	5.1	2102	104	31	NA	50.0	18.5	18.8	74.9	3.1	5.8
ASCOT-LLA [97],2003	S,b,PR	3.2	10305	210	NA	NA	63.0	32.7	24.6	100.0	0.0	–
Mohler [98],2003	S,b,SE	1	354	2	1	1	68.0	40.4	17.5	–	–	–
ALLIANCE [99],2004	S,b,SE	4.3	2442	74	NA	NA	61.2	19.5	22.1	–	57.8	6.6
ARBITER2 [100],2004	O,b,SE	*1*	167	1	NA	NA	67.5	10.2	27.5	74.9	49.7	–
BaeJH [101],2004	S,b,SE	*6*	205	2	0	2	60.0	41.5	29.8	48.3	12.2	–
CARDS [102],2004	S,b,PR	3.9	2838	60	6	50	62.0	22.2	100.0	83.8	0.0	0.0
PCS [103],2004	S,b,SE	5	120	7	NA	NA	59.6	67.5	17.5	59.2	–	–
4D [104], [105],2005	S,b,SE	4	1255	103	40	65	65.7	8.6	100.0	–	17.6	–
FIELD [106], [107],2005	F,b,SE	*5*	9795	333	NA	NA	62.2	9.4	100.0	56.6	5.0	3.5
Makuuchi [108],2005	S,op,SE	4.5	303	6	1	NA	58.9	41.9	33.3	51.5	62.0	–
Stone [109],2005	S,b,SE	*1*	300	2	NA	NA	–	0.0	16.0	63.6	39.3	–
ASPEN prim [110],2006	S,b,PR	4	1905	56	NA	NA	60.5	13.2	100.0	52.3	0.0	–
ASPEN sec [110],2006	S,b,SE	4	505	16	NA	NA	63.2	9.7	100.0	65.5	78.2	–
SPARCL [111],2006	S,b,SE	4.9	4731	576	65	527	62.7	19.2	16.7	61.9	30.9	69.1
WHI-DM [112],2006	D,o,PS	8.1	48835	1076	150	935	62.3	6.7	–	42.9	1.9	1.1
CORONA ,[120],2007	S,b,SE	2.7	5011	NA	67	197	73.0	8.6	29.5	63.4	59.9	12.4
ARISE [113],2008	O,b,SE	2	6144	54	0	64	65.0	13.5	37.0	72.0	72.0	–
CCSPS [114],2008	O,b,SE	4.5	4870	NA	25	NA	58.9	34.5	12.5	55.5	100.0	–
GISSI-HF [115],2008	S,b,SE	3.9	4574	148	67	86	68.0	14.1	26.1	54.3	–	4.5
JUPITER [116], [117],2008	S,b,PR	1.9	17802	97	NA	88	66.0	15.8	0.0	57.3	0.0	0.0
OACIS lipid [118],2008	S,o,SE	0.7	353	2	NA	NA	63.2	57.4	31.7	747.6	100.0	7.3
**IMPROVE-IT**[119]**,2015**	O,b,SE	6	18.144	641	NA	NA	64.0	33.0	27.2	61.5	21.0	NA

**AGE** mean age; **SMK** smoking status; **DM** diabetes mellitus; **HBP** high blood pressure; **PMI** previous myocardial infarction; **PST** previous stroke.

a **DESIGN**: the first letter indicates the type of lipid lowering intervention (D: diet, S; statins, F: fibrates, O: other drugs, B: ileal bypass or other surgery); the second letter indicates the study design (op: open; b blind); the last letter indicates the clinical setting (PR: primary, SE: secondary, PS: primary and secondary)

b **FOLLOW-UP** indicates mean duration (year), in its absence the maximum follow-up duration is indicated *(in italics)*;

## References

[1] Di Napoli P., Taccardi A.A., Oliver M., De Caterina R. (2002). Statins and stroke: evidence for cholesterol-independent effects. Eur. Heart J..

[2] De Caterina R., Scarano M., Marfisi R., Lucisano G., Palma F., Tatasciore A., Marchioli R. (2010). Cholesterol-lowering interventions and stroke: insights from a meta-analysis of randomized controlled trials. J. Am. Coll. Cardiol..

[3] Cannon C.P., Blazing M.A., Giugliano R.P., McCagg A., White J.A., Theroux P., Darius H., Lewis B.S., Ophuis T.O., Jukema J.W., De Ferrari G.M., Ruzyllo W., De Lucca P., Im K., Bohula E.A., Reist C., Wiviott S.D., Tershakovec A.M., Musliner T.A., Braunwald E., Califf R.M. (2015). Ezetimibe added to statin therapy after acute coronary syndromes. New Engl. J. Med..

[4] De Caterina R., Salvatore T., Marchioli R. (2016). Cholesterol-lowering interventions and stroke: insights from IMPROVE-IT. Atherosclerosis.

[5] De Caterina R., Salvatore T., Marchioli R. (2016). Cholesterol-lowering interventions and stroke: insights from IMPROVE-IT. Atherosclerosis.

[6] Leren P. (1970). The Oslo diet-heart study. Eleven-year report.

[7] Ederer F., Leren P., Turpeinen O., Frantz Jr. I.D. (1971). Cancer among men on cholesterol-lowering diets. Experience from five clinical trials. Lancet.

[8] Pearce M.L., Dayton S. (1971). Incidence of cancer in men on a diet high in polyunsaturated fat. Lancet.

[9] Leren P. (1966). The effect of plasma cholesterol lowering diet in male survivors of myocardial infarction. A controlled clinical trial. Acta Med. Scand. Suppl..

[10] (1968). Controlled trial of soya-bean oil in myocardial infarction. Lancet.

[11] Dayton S., Pearce M.L., Hashimoto S., Dixon W.J., Tomiyasu U. (1969). A controlled clinical trial of a diet high in unsaturated fat in preventing complications of atherosclerosis. Circulation.

[12] The Newcastle upon Tyne Region Study Group (1971). Trial of clofibrate in the treatment of ischaemic heart disease. Five-year study by a group of physicians of the Newcastle upon Tyne region. Br. Med. J..

[13] (1971). Ischaemic heart disease: a secondary prevention trial using clofibrate. Report by a research committee of the Scottish Society of Physicians. Br. Med. J..

[14] Dewar H.A., Oliver M.F. (1972). Trial of clofibrate. Br. Med. J..

[15] (1973). The treatment of cerebrovascular disease with clofibrate. Final report of the Veterans Administration Cooperative Study of Atherosclerosis, Neurology Section. Stroke.

[16] The Coronary Drug Project (1970). Initial findings leading to modifications of its research protocol. JAMA.

[17] (1975). Clofibrate and niacin in coronary heart disease. JAMA.

[18] Coronary Drug Project Research Group (1978). Natural history of myocardial infarction in the coronary drug project: long-term prognostic importance of serum lipid levels. Am. J. Cardiol..

[19] Canner P.L., Berge K.G., Wenger N.K., Stamler J., Friedman L., Prineas R.J., Friedewald W. (1986). Fifteen year mortality in coronary drug project patients: long-term benefit with niacin. J. Am. Coll. Cardiol..

[20] Dorr A.E., Gundersen K., Schneider J.C., Spencer T.W., Martin W.B. (1978). Colestipol hydrochloride in hypercholesterolemic patients-effect on serum cholesterol and mortality. J. Chronic Dis..

[21] (1978). A co-operative trial in the primary prevention of ischaemic heart disease using clofibrate. Report from the Committee of Principal Investigators. Br. Heart J..

[22] (1980). WHO cooperative trial on primary prevention of ischaemic heart disease using clofibrate to lower serum cholesterol: mortality follow-up. Report of the Committee of Principal Investigators. Lancet.

[23] (1984). WHO cooperative trial on primary prevention of ischaemic heart disease with clofibrate to lower serum cholesterol: final mortality follow-up. Report of the Committee of Principal Investigators. Lancet.

[24] Heady J.A., Morris J.N., Oliver M.F. (1992). WHO clofibrate/cholesterol trial: clarifications. Lancet.

[25] McCaughan D. (1981). The long-term effects of probucol on serum lipid levels. Arch. Intern. Med..

[26] (1984). The Lipid Research Clinics Coronary Primary Prevention Trial Results. I. Reduction in incidence of coronary heart disease. JAMA.

[27] Blankenhorn D.H., Nessim S.A., Johnson R.L., Sanmarco M.E., Azen S.P., Cashin-Hemphill L. (1987). Beneficial effects of combined colestipol-niacin therapy on coronary atherosclerosis and coronary venous bypass grafts. JAMA.

[28] Cashin-Hemphill L., Mack W.J., Pogoda J.M., Sanmarco M.E., Azen S.P., Blankenhorn D.H. (1990). Beneficial effects of colestipol-niacin on coronary atherosclerosis. A 4-year follow-up. JAMA.

[29] Frick M.H., Elo O., Haapa K., Heinonen O.P., Heinsalmi P., Helo P., Huttunen J.K., Kaitaniemi P., Koskinen P., Manninen V. (1987). Helsinki Heart Study: primary-prevention trial with gemfibrozil in middle-aged men with dyslipidemia. Safety of treatment, changes in risk factors, and incidence of coronary heart disease. New Engl. J. Med..

[30] Manninen V., Elo M.O., Frick M.H., Haapa K., Heinonen O.P., Heinsalmi P., Helo P., Huttunen J.K., Kaitaniemi P., Koskinen P. (1988). Lipid alterations and decline in the incidence of coronary heart disease in the Helsinki Heart Study. JAMA.

[31] Carlson L.A., Danielson M., Ekberg I., Klintemar B., Rosenhamer G. (1977). Reduction of myocardial reinfarction by the combined treatment with clofibrate and nicotinic acid. Atherosclerosis.

[32] Carlson L.A., Rosenhamer G. (1988). Reduction of mortality in the Stockholm ischaemic Heart Disease Secondary Prevention Study by combined treatment with clofibrate and nicotinic acid. Acta Med. Scand..

[33] Frantz I.D., Dawson E.A., Ashman P.L., Gatewood L.C., Bartsch G.E., Kuba K., Brewer E.R. (1989). Test of effect of lipid lowering by diet on cardiovascular risk. The Minnesota Coronary Survey. Atherosclerosis.

[34] Brown G., Albers J.J., Fisher L.D., Schaefer S.M., Lin J.T., Kaplan C., Zhao X.Q., Bisson B.D., Fitzpatrick V.F., Dodge H.T. (1990). Regression of coronary artery disease as a result of intensive lipid-lowering therapy in men with high levels of apolipoprotein B. New Engl. J. Med..

[35] Buchwald H., Matts J.P., Hansen B.J., Long J.M., Fitch L.L. (1987). Program on surgical control of the hyperlipidemias (POSCH): recruitment experience. Control. Clin. Trials.

[36] Buchwald H., Matts J.P., Fitch L.L., Varco R.L., Campbell G.S., Pearce M., Yellin A., Smink R.D., Sawin H.S., Campos C.T. (1989). Program on the Surgical Control of the Hyperlipidemias (POSCH): design and methodology. POSCH Group. J. Clin. Epidemiol..

[37] Buchwald H., Varco R.L., Matts J.P., Long J.M., Fitch L.L., Campbell G.S., Pearce M.B., Yellin A.E., Edmiston W.A., Smink R.D. (1990). Effect of partial ileal bypass surgery on mortality and morbidity from coronary heart disease in patients with hypercholesterolemia. Report of the Program on the Surgical Control of the Hyperlipidemias (POSCH). New Engl. J. Med..

[38] Buchwald H., Matts J.P., Fitch L.L., Campos C.T., Sanmarco M.E., Amplatz K., Castaneda-Zuniga W.R., Hunter D.W., Pearce M.B., Bissett J.K. (1992). Changes in sequential coronary arteriograms and subsequent coronary events. Surgical Control of the Hyperlipidemias (POSCH) Group. JAMA.

[39] Bradford R.H., Shear C.L., Chremos A.N., Dujovne C., Franklin F.A., Hesney M., Higgins J., Langendorfer A., Pool J.L., Schnaper H. (1990). Expanded Clinical Evaluation of Lovastatin (EXCEL) study: design and patient characteristics of a double-blind, placebo-controlled study in patients with moderate hypercholesterolemia. Am. J. Cardiol..

[40] Bradford R.H., Shear C.L., Chremos A.N., Dujovne C., Downton M., Franklin F.A., Gould A.L., Hesney M., Higgins J., Hurley D.P. (1991). Expanded Clinical Evaluation of Lovastatin (EXCEL) study results. I. Efficacy in modifying plasma lipoproteins and adverse event profile in 8245 patients with moderate hypercholesterolemia. Arch. Intern. Med..

[41] Tobert J.A. (1992). The cholesterol controversy. BMJ.

[42] Singh R.B., Rastogi S.S., Verma R., Bolaki L., Singh R. (1992). An Indian experiment with nutritional modulation in acute myocardial infarction. Am. J. Cardiol..

[43] Singh R.B., Rastogi S.S., Verma R., Laxmi B., Singh R., Ghosh S., Niaz M.A. (1992). Randomised controlled trial of cardioprotective diet in patients with recent acute myocardial infarction: results of one year follow up. BMJ.

[44] Frick M.H., Heinonen O.P., Huttunen J.K., Koskinen P., Manttari M., Manninen V. (1993). Efficacy of gemfibrozil in dyslipidaemic subjects with suspected heart disease. An ancillary study in the Helsinki Heart Study frame population. Ann. Med..

[45] Blankenhorn D.H., Azen S.P., Kramsch D.M., Mack W.J., Cashin-Hemphill L., Hodis H.N., DeBoer L.W., Mahrer P.R., Masteller M.J., Vailas L.I., Alaupovic P., Hirsch L.J. (1993). Coronary angiographic changes with lovastatin therapy. The Monitored Atherosclerosis Regression Study (MARS). Ann. Intern Med..

[46] Hodis H.N., Mack W.J., Azen S.P., Alaupovic P., Pogoda J.M., LaBree L., Hemphill L.C., Kramsch D.M., Blankenhorn D.H. (1994). Triglyceride- and cholesterol-rich lipoproteins have a differential effect on mild/moderate and severe lesion progression as assessed by quantitative coronary angiography in a controlled trial of lovastatin. Circulation.

[47] (1993). Effects of pravastatin in patients with serum total cholesterol levels from 5.2 to 7.8 mmol/liter (200–300 mg/dl) plus two additional atherosclerotic risk factors. The Pravastatin Multinational Study Group for Cardiac Risk Patients. Am. J. Cardiol..

[48] (1993). Design and baseline results of the Scandinavian Simvastatin Survival Study of patients with stable angina and/or previous myocardial infarction. Am. J. Cardiol..

[49] (1994). Randomised trial of cholesterol lowering in 4444 patients with coronary heart disease: the Scandinavian Simvastatin Survival Study (4S). Lancet.

[50] Pedersen T.R., Olsson A.G., Faergeman O., Kjekshus J., Wedel H., Berg K., Wilhelmsen L., Haghfelt T., Thorgeirsson G., Pyorala K., Miettinen T., Christophersen B., Tobert J.A., Musliner T.A., Cook T.J. (1998). Lipoprotein changes and reduction in the incidence of major coronary heart disease events in the Scandinavian Simvastatin Survival Study (4S). Circulation.

[51] Furberg C.D., Adams H.P., Applegate W.B., Byington R.P., Espeland M.A., Hartwell T., Hunninghake D.B., Lefkowitz D.S., Probstfield J., Riley W.A. (1994). Effect of lovastatin on early carotid atherosclerosis and cardiovascular events. Asymptomatic Carotid Artery Progression Study (ACAPS) Research Group. Circulation.

[52] Waters D., Higginson L., Gladstone P., Kimball B., Le May M., Boccuzzi S.J., Lesperance J. (1994). Effects of monotherapy with an HMG-CoA reductase inhibitor on the progression of coronary atherosclerosis as assessed by serial quantitative arteriography. The Canadian Coronary Atherosclerosis Intervention Trial. Circulation.

[53] Weintraub W.S., Boccuzzi S.J., Klein J.L., Kosinski A.S., King S.B., Ivanhoe R., Cedarholm J.C., Stillabower M.E., Talley J.D., DeMaio S.J. (1994). Lack of effect of lovastatin on restenosis after coronary angioplasty. Lovastatin Restenosis Trial Study Group. New Engl. J. Med..

[54] de Lorgeril M., Renaud S., Mamelle N., Salen P., Martin J.L., Monjaud I., Guidollet J., Touboul P., Delaye J. (1994). Mediterranean alpha-linolenic acid-rich diet in secondary prevention of coronary heart disease. Lancet.

[55] Pitt B., Ellis S.G., Mancini G.B., Rosman H.S., McGovern M.E. (1993). Design and recruitment in the United States of a multicenter quantitative angiographic trial of Pravastatin to Limit Atherosclerosis in the Coronary Arteries (PLAC I). Am. J. Cardiol..

[56] Furberg C.D., Byington R.P., Crouse J.R., Espeland M.A. (1994). Pravastatin, lipids, and major coronary events. Am. J. Cardiol..

[57] Furberg C.D., Pitt B., Byington R.P., Park J.S., McGovern M.E. (1995). Reduction in coronary events during treatment with pravastatin. PLAC I and PLAC II Investigators. Pravastatin Limitation of Atherosclerosis in the Coronary Arteries. Am. J. Cardiol..

[58] Pitt B., Mancini G.B., Ellis S.G., Rosman H.S., Park J.S., McGovern M.E. (1995). Pravastatin Limitation of Atherosclerosis in the Coronary Arteries (PLAC I): reduction in atherosclerosis progression and clinical events. PLAC I Investigation. J. Am. Coll. Cardiol..

[59] Furberg C.D., Crouse J.R., Byington R.P., Bond M.G., Espeland M.A. (1993). PLAC-2: effects of pravastatin on progression of carotid atherosclerosis and clinical events (Abstract 716-5). J. Am. Coll. Cardiol..

[60] Crouse J.R., Byington R.P., Bond M.G., Espeland M.A., Craven T.E., Sprinkle J.W., McGovern M.E., Furberg C.D. (1995). Pravastatin, Lipids, and Atherosclerosis in the Carotid Arteries (PLAC-II). Am. J. Cardiol..

[61] de Groot E., Jukema J.W., van Boven A.J., Reiber J.H., Zwinderman A.H., Lie K.I., Ackerstaff R.A., Bruschke A.V. (1995). Effect of pravastatin on progression and regression of coronary atherosclerosis and vessel wall changes in carotid and femoral arteries: a report from the Regression Growth Evaluation Statin Study. Am. J. Cardiol..

[62] Jukema J.W., Bruschke A.V., van Boven A.J., Reiber J.H., Bal E.T., Zwinderman A.H., Jansen H., Boerma G.J., van Rappard F.M., Lie K.I. (1995). Effects of lipid lowering by pravastatin on progression and regression of coronary artery disease in symptomatic men with normal to moderately elevated serum cholesterol levels. The Regression Growth Evaluation Statin Study (REGRESS). Circulation.

[63] Salonen R., Nyyssonen K., Porkkala E., Rummukainen J., Belder R., Park J.S., Salonen J.T. (1995). Kuopio Atherosclerosis Prevention Study (KAPS). A population-based primary preventive trial of the effect of LDL lowering on atherosclerotic progression in carotid and femoral arteries. Circulation.

[64] Sacks F.M., Pfeffer M.A., Moye L.A., Rouleau J.L., Rutherford J.D., Cole T.G., Brown L., Warnica J.W., Arnold J.M., Wun C.C., Davis B.R., Braunwald E. (1996). The effect of pravastatin on coronary events after myocardial infarction in patients with average cholesterol levels. Cholesterol and Recurrent Events Trial Investigators. New Engl. J. Med..

[65] Sacks F.M., Moye L.A., Davis B.R., Cole T.G., Rouleau J.L., Nash D.T., Pfeffer M.A., Braunwald E. (1998). Relationship between plasma LDL concentrations during treatment with pravastatin and recurrent coronary events in the Cholesterol and Recurrent Events Trial. Circulation.

[66] Shepherd J., Cobbe S.M., Ford I., Isles C.G., Lorimer A.R., MacFarlane P.W., McKillop J.H., Packard C.J. (1995). Prevention of coronary heart disease with pravastatin in men with hypercholesterolemia. West of Scotland Coronary Prevention Study Group. New Engl. J. Med..

[67] Bestehorn H.P., Rensing U.F., Roskamm H., Betz P., Benesch L., Schemeitat K., Blumchen G., Claus J., Mathes P., Kappenberger L., Wieland H., Neiss A. (1997). The effect of simvastatin on progression of coronary artery disease. The Multicenter Coronary Intervention Study (CIS). Eur. Heart J..

[68] Frick M.H., Syvanne M., Nieminen M.S., Kauma H., Majahalme S., Virtanen V., Kesaniemi Y.A., Pasternack A., Taskinen M.R. (1997). Prevention of the angiographic progression of coronary and vein-graft atherosclerosis by gemfibrozil after coronary bypass surgery in men with low levels of HDL cholesterol. Lopid Coronary Angiography Trial (LOCAT) Study Group. Circulation.

[69] Post Coronary Artery Bypass Graft Trial Investigators (1997). The effect of aggressive lowering of low-density lipoprotein cholesterol levels and low-dose anticoagulation on obstructive changes in saphenous-vein coronary-artery bypass grafts. The Post Coronary Artery Bypass Graft Trial Investigators. New Engl. J. Med..

[70] Bertrand M.E., McFadden E.P., Fruchart J.C., Van Belle E., Commeau P., Grollier G., Bassand J.P., Machecourt J., Cassagnes J., Mossard J.M., Vacheron A., Castaigne A., Danchin N., Lablanche J.M. (1997). Effect of pravastatin on angiographic restenosis after coronary balloon angioplasty. The PREDICT trial investigators. Prevention of Restenosis by Elisor after Transluminal Coronary Angioplasty. J. Am. Coll. Cardiol..

[71] Downs J.R., Clearfield M., Weis S., Whitney E., Shapiro D.R., Beere P.A., Langendorfer A., Stein E.A., Kruyer W., Gotto A.M. (1998). Primary prevention of acute coronary events with lovastatin in men and women with average cholesterol levels: results of AFCAPS/TexCAPS. Air Force/Texas Coronary Atherosclerosis Prevention Study. JAMA.

[72] (1998). The Long-Term Intervention with Pravastatin in Ischaemic Disease (lipid) Study Group. Prevention of cardiovascular events and death with pravastatin in patients with coronary heart disease and a broad range of initial cholesterol levels. The Long-Term Intervention with Pravastatin in Ischaemic Disease (LIPID) Study Group. New Engl. J. Med..

[73] White H.D., Simes R.J., Anderson N.E., Hankey G.J., Watson J.D., Hunt D., Colquhoun D.M., Glasziou P., MacMahon S., Kirby A.C., West M.J., Tonkin A.M. (2000). Pravastatin therapy and the risk of stroke. New Engl. J. Med..

[74] LIPID Study Group (2002). Long-term effectiveness and safety of pravastatin in 9014 patients with coronary heart disease and average cholesterol concentrations: the LIPID trial follow-up. Lancet.

[75] Mas R., Castano G., Illnait J., Fernandez L., Fernandez J., Aleman C., Pontigas V., Lescay M. (1999). Effects of policosanol in patients with type II hypercholesterolemia and additional coronary risk factors. Clin. Pharmacol. Ther..

[76] GISSI Prevenzione Investigators (2000). Results of the low-dose (20 mg) pravastatin GISSI Prevenzione trial in 4271 patients with recent myocardial infarction: do stopped trials contribute to overall knowledge?. Ital. Heart J..

[77] Teo K.K., Burton J.R., Buller C.E., Plante S., Catellier D., Tymchak W., Dzavik V., Taylor D., Yokoyama S., Montague T.J. (2000). Long-term effects of cholesterol lowering and angiotensin-converting enzyme inhibition on coronary atherosclerosis: The Simvastatin/Enalapril Coronary Atherosclerosis Trial (SCAT). Circulation.

[78] Bloomfield Rubins H., Davenport J., Babikian V., Brass L.M., Collins D., Wexler L., Wagner S., Papademetriou V., Rutan G., Robins S.J. (2001). Reduction in stroke with gemfibrozil in men with coronary heart disease and low HDL cholesterol: The Veterans Affairs HDL Intervention Trial (VA-HIT). Circulation.

[79] Hedblad B., Wikstrand J., Janzon L., Wedel H., Berglund G. (2001). Low-dose metoprolol CR/XL and fluvastatin slow progression of carotid intima-media thickness: main results from the Beta-Blocker Cholesterol-Lowering Asymptomatic Plaque Study (BCAPS). Circulation.

[80] The BIP Study Group (2000). Secondary prevention by raising HDL cholesterol and reducing triglycerides in patients with coronary artery disease: the Bezafibrate Infarction Prevention (BIP) study. Circulation.

[81] Steiner G., Stewart D., Hosking J.D. (1999). Baseline characteristics of the study population in the Diabetes Atherosclerosis Intervention Study (DAIS). World Health Organization Collaborating Centre for the Study of Atherosclerosis in Diabetes. Am. J. Cardiol..

[82] (2001). Effect of fenofibrate on progression of coronary-artery disease in type 2 diabetes: the Diabetes Atherosclerosis Intervention Study, a randomised study. Lancet.

[83] Vakkilainen J., Steiner G., Ansquer J.C., Aubin F., Rattier S., Foucher C., Hamsten A., Taskinen M.R. (2003). Relationships between low-density lipoprotein particle size, plasma lipoproteins, and progression of coronary artery disease: the Diabetes Atherosclerosis Intervention Study (DAIS). Circulation.

[84] Brown B.G., Zhao X.Q., Chait A., Fisher L.D., Cheung M.C., Morse J.S., Dowdy A.A., Marino E.K., Bolson E.L., Alaupovic P., Frohlich J., Albers J.J. (2001). Simvastatin and niacin, antioxidant vitamins, or the combination for the prevention of coronary disease. New Engl. J. Med..

[85] ALLHAT Collaborative Research Group (2002). Major outcomes in moderately hypercholesterolemic, hypertensive patients randomized to pravastatin vs usual care: The Antihypertensive and Lipid-Lowering Treatment to Prevent Heart Attack Trial (ALLHAT-LLT). JAMA.

[86] Sawayama Y., Shimizu C., Maeda N., Tatsukawa M., Kinukawa N., Koyanagi S., Kashiwagi S., Hayashi J. (2002). Effects of probucol and pravastatin on common carotid atherosclerosis in patients with asymptomatic hypercholesterolemia. Fukuoka Atherosclerosis Trial (FAST). J Am. Coll. Cardiol..

[87] Athyros V.G., Papageorgiou A.A., Mercouris B.R., Athyrou V.V., Symeonidis A.N., Basayannis E.O., Demitriadis D.S., Kontopoulos A.G. (2002). Treatment with atorvastatin to the National Cholesterol Educational Program goal versus ׳usual׳ care in secondary coronary heart disease prevention. The GREek Atorvastatin and Coronary-Heart-Disease Evaluation (GREACE) study. Curr. Med. Res. Opin..

[88] Heart Protection Study Collaborative Group (2002). MRC/BHF Heart Protection Study of cholesterol lowering with simvastatin in 20,536 high-risk individuals: a randomised placebo-controlled trial. Lancet.

[89] Emberson J.R., Ng L.L., Armitage J., Bowman L., Parish S., Collins R. (2007). N-terminal Pro-B-type natriuretic peptide, vascular disease risk, and cholesterol reduction among 20,536 patients in the MRC/BHF heart protection study. J. Am. Coll. Cardiol..

[90] Meade T.W. (2001). Design and intermediate results of the Lower Extremity Arterial Disease Event Reduction (LEADER)* trial of bezafibrate in men with lower extremity arterial disease (ISRCTN4119421). Current Control. Trials Cardiovasc. Med..

[91] Meade T., Zuhrie R., Cook C., Cooper J. (2002). Bezafibrate in men with lower extremity arterial disease: randomised controlled trial. BMJ.

[92] Liem A.H., van Boven A.J., Veeger N.J., Withagen A.J., Robles de Medina R.M., Tijssen J.G., van Veldhuisen D.J. (2002). Effect of fluvastatin on ischaemia following acute myocardial infarction: a randomized trial. Eur. Heart J..

[93] Serruys P.W., de Feyter P., Macaya C., Kokott N., Puel J., Vrolix M., Branzi A., Bertolami M.C., Jackson G., Strauss B., Meier B. (2002). Fluvastatin for prevention of cardiac events following successful first percutaneous coronary intervention: a randomized controlled trial. JAMA.

[94] Shepherd J., Blauw G.J., Murphy M.B., Bollen E.L., Buckley B.M., Cobbe S.M., Ford I., Gaw A., Hyland M., Jukema J.W., Kamper A.M., Macfarlane P.W., Meinders A.E., Norrie J., Packard C.J., Perry I.J., Stott D.J., Sweeney B.J., Twomey C., Westendorp R.G. (2002). Pravastatin in elderly individuals at risk of vascular disease (PROSPER): a randomised controlled trial. Lancet.

[95] Holdaas H., Fellstrom B., Jardine A.G., Holme I., Nyberg G., Fauchald P., Gronhagen-Riska C., Madsen S., Neumayer H.H., Cole E., Maes B., Ambuhl P., Olsson A.G., Hartmann A., Solbu D.O., Pedersen T.R. (2003). Effect of fluvastatin on cardiac outcomes in renal transplant recipients: a multicentre, randomised, placebo-controlled trial. Lancet.

[96] Holdaas H., Fellstrom B., Jardine A.G., Nyberg G., Gronhagen-Riska C., Madsen S., Neumayer H.H., Cole E., Maes B., Ambuhl P., Logan J.O., Staffler B., Gimpelewicz C. (2005). Beneficial effect of early initiation of lipid-lowering therapy following renal transplantation. Nephrol. Dial. Transplant..

[97] Sever P.S., Dahlof B., Poulter N.R., Wedel H., Beevers G., Caulfield M., Collins R., Kjeldsen S.E., Kristinsson A., McInnes G.T., Mehlsen J., Nieminen M., O׳Brien E., Ostergren J. (2003). Prevention of coronary and stroke events with atorvastatin in hypertensive patients who have average or lower-than-average cholesterol concentrations, in the Anglo-Scandinavian Cardiac Outcomes Trial—Lipid Lowering Arm (ASCOT-LLA): a multicentre randomised controlled trial. Lancet.

[98] Mohler E.R., Hiatt W.R., Creager M.A. (2003). Cholesterol reduction with atorvastatin improves walking distance in patients with peripheral arterial disease. Circulation.

[99] Koren M.J., Hunninghake D.B. (2004). Clinical outcomes in managed-care patients with coronary heart disease treated aggressively in lipid-lowering disease management clinics: the alliance study. J. Am. Coll. Cardiol..

[100] Taylor A.J., Sullenberger L.E., Lee H.J., Lee J.K., Grace K.A. (2004). Arterial Biology for the Investigation of the Treatment Effects of Reducing Cholesterol (ARBITER) 2: a double-blind, placebo-controlled study of extended-release niacin on atherosclerosis progression in secondary prevention patients treated with statins. Circulation.

[101] Bae J.H., Bassenge E., Kim K.Y., Synn Y.C., Park K.R., Schwemmer M. (2004). Effects of low-dose atorvastatin on vascular responses in patients undergoing percutaneous coronary intervention with stenting. J. Cardiovasc. Pharmacol. Ther..

[102] Colhoun H.M., Betteridge D.J., Durrington P.N., Hitman G.A., Neil H.A., Livingstone S.J., Thomason M.J., Mackness M.I., Charlton-Menys V., Fuller J.H. (2004). Primary prevention of cardiovascular disease with atorvastatin in type 2 diabetes in the Collaborative Atorvastatin Diabetes Study (CARDS): multicentre randomised placebo-controlled trial. Lancet.

[103] Nakagawa T., Kobayashi T., Awata N., Sato S., Reiber J.H., Nakajima H., Toyama Y.N., Hiraoka H., Kato O., Kirino M., Kobayashi T., Takeda Y., Yachiku K., Iida M., Itoh T., Shibata N. (2004). Randomized, controlled trial of secondary prevention of coronary sclerosis in normocholesterolemic patients using pravastatin: final 5-year angiographic follow-up of the Prevention of Coronary Sclerosis (PCS) study. Int. J. Cardiol..

[104] Wanner C., Krane V., Marz W., Olschewski M., Asmus H.G., Kramer W., Kuhn K.W., Kutemeyer H., Mann J.F., Ruf G., Ritz E. (2004). Randomized controlled trial on the efficacy and safety of atorvastatin in patients with type 2 diabetes on hemodialysis (4D study): demographic and baseline characteristics. Kidney Blood Press. Res..

[105] Wanner C., Krane V., Marz W., Olschewski M., Mann J.F., Ruf G., Ritz E. (2005). Atorvastatin in patients with type 2 diabetes mellitus undergoing hemodialysis. New Engl. J. Med..

[106] Keech A., Simes R.J., Barter P., Best J., Scott R., Taskinen M.R., Forder P., Pillai A., Davis T., Glasziou P., Drury P., Kesaniemi Y.A., Sullivan D., Hunt D., Colman P., d׳Emden M., Whiting M., Ehnholm C., Laakso M. (2005). Effects of long-term fenofibrate therapy on cardiovascular events in 9795 people with type 2 diabetes mellitus (the FIELD study): randomised controlled trial. Lancet.

[107] Scott R., Best J., Forder P., Taskinen M.R., Simes J., Barter P., Keech A. (2005). Fenofibrate Intervention and Event Lowering in Diabetes (FIELD) study: baseline characteristics and short-term effects of fenofibrate (ISRCTN64783481). Cardiovasc. Diabetol..

[108] Makuuchi H., Furuse A., Endo M., Nakamura H., Daida H., Watanabe M., Ohashi Y., Hosoda Y., Hosoda S., Yamaguchi H., Yasui H. (2005). Effect of pravastatin on progression of coronary atherosclerosis in patients after coronary artery bypass surgery. Circ. J..

[109] Stone P.H., Lloyd-Jones D.M., Kinlay S., Frei B., Carlson W., Rubenstein J., Andrews T.C., Johnstone M., Sopko G., Cole H., Orav J., Selwyn A.P., Creager M.A. (2005). Effect of intensive lipid lowering, with or without antioxidant vitamins, compared with moderate lipid lowering on myocardial ischemia in patients with stable coronary artery disease: the Vascular Basis for the Treatment of Myocardial Ischemia Study. Circulation.

[110] Knopp R.H., d׳Emden M., Smilde J.G., Pocock S.J. (2006). Efficacy and safety of atorvastatin in the prevention of cardiovascular end points in subjects with type 2 diabetes: the Atorvastatin Study for Prevention of Coronary Heart Disease Endpoints in non-insulin-dependent diabetes mellitus (ASPEN). Diabetes Care.

[111] Amarenco P., Bogousslavsky J., Callahan A., Goldstein L.B., Hennerici M., Rudolph A.E., Sillesen H., Simunovic L., Szarek M., Welch K.M., Zivin J.A. (2006). High-dose atorvastatin after stroke or transient ischemic attack. New Engl. J. Med..

[112] Howard B.V., Van Horn L., Hsia J., Manson J.E., Stefanick M.L., Wassertheil-Smoller S., Kuller L.H., LaCroix A.Z., Langer R.D., Lasser N.L., Lewis C.E., Limacher M.C., Margolis K.L., Mysiw W.J., Ockene J.K., Parker L.M., Perri M.G., Phillips L., Prentice R.L., Robbins J., Rossouw J.E., Sarto G.E., Schatz I.J., Snetselaar L.G., Stevens V.J., Tinker L.F., Trevisan M., Vitolins M.Z., Anderson G.L., Assaf A.R., Bassford T., Beresford S.A., Black H.R., Brunner R.L., Brzyski R.G., Caan B., Chlebowski R.T., Gass M., Granek I., Greenland P., Hays J., Heber D., Heiss G., Hendrix S.L., Hubbell F.A., Johnson K.C., Kotchen J.M. (2006). Low-fat dietary pattern and risk of cardiovascular disease: the women׳s health initiative randomized controlled dietary modification trial. JAMA.

[113] Tardif J.C., McMurray J.J., Klug E., Small R., Schumi J., Choi J., Cooper J., Scott R., Lewis E.F., L׳Allier P.L., Pfeffer M.A. (2008). Effects of succinobucol (AGI-1067) after an acute coronary syndrome: a randomised, double-blind, placebo-controlled trial. Lancet.

[114] Lu Z., Kou W., Du B., Wu Y., Zhao S., Brusco O.A., Morgan J.M., Capuzzi D.M., Li S. (2008). Effect of Xuezhikang, an extract from red yeast Chinese rice, on coronary events in a Chinese population with previous myocardial infarction. Am. J. Cardiol..

[115] Tavazzi L., Maggioni A.P., Marchioli R., Barlera S., Franzosi M.G., Latini R., Lucci D., Nicolosi G.L., Porcu M., Tognoni G. (2008). Effect of rosuvastatin in patients with chronic heart failure (the GISSI-HF trial): a randomised, double-blind, placebo-controlled trial. Lancet.

[116] Ridker P.M., Fonseca F.A., Genest J., Gotto A.M., Kastelein J.J., Khurmi N.S., Koenig W., Libby P., Lorenzatti A.J., Nordestgaard B.G., Shepherd J., Willerson J.T., Glynn R.J. (2007). Baseline characteristics of participants in the JUPITER trial, a randomized placebo-controlled primary prevention trial of statin therapy among individuals with low low-density lipoprotein cholesterol and elevated high-sensitivity C-reactive protein. Am. J. Cardiol..

[117] Ridker P.M., Danielson E., Fonseca F.A., Genest J., Gotto A.M., Kastelein J.J., Koenig W., Libby P., Lorenzatti A.J., MacFadyen J.G., Nordestgaard B.G., Shepherd J., Willerson J.T., Glynn R.J. (2008). Rosuvastatin to prevent vascular events in men and women with elevated C-reactive protein. New Engl. J. Med..

[118] Sato H., Kinjo K., Ito H., Hirayama A., Nanto S., Fukunami M., Nishino M., Lim Y.J., Kijima Y., Koretsune Y., Nakatani D., Mizuno H., Shimizu M., Hori M. (2008). Effect of early use of low-dose pravastatin on major adverse cardiac events in patients with acute myocardial infarction: the OACIS-LIPID Study. Circ. J..

[119] Cannon C.P., Blazing M.A., Giugliano R.P., McCagg A., White J.A., Theroux P., Darius H., Lewis B.S., Ophuis T.O., Jukema J.W., De Ferrari G.M., Ruzyllo W., De Lucca P., Im K., Bohula E.A., Reist C., Wiviott S.D., Tershakovec A.M., Musliner T.A., Braunwald E., Califf R.M. (2015). Ezetimibe Added to Statin Therapy after Acute Coronary Syndromes. New Engl. J. Med..

[120] Kjekshus J., Apetrei E., Barrios V., Bohm M., Cleland J.G., Cornel J.H., Dunselman P., Fonseca C., Goudev A., Grande P., Gullestad L., Hjalmarson A., Hradec J., Janosi A., Kamensky G., Komajda M., Korewicki J., Kuusi T., Mach F., Mareev V., McMurray J.J., Ranjith N., Schaufelberger M., Vanhaecke J., van Veldhuisen D.J., Waagstein F., Wedel H., Wikstrand J. (2007). Rosuvastatin in older patients with systolic heart failure.. N Engl J Med.

