# Supplementary material for: All cholesterol-lowering interventions are expected to reduce stroke: Confirmatory data from IMPROVE-IT
Source: Data Brief. 2016 Apr 27;7:1541–50. doi: 10.1016/j.dib.2016.04.059 (PMC4865673; doi:10.1016/j.dib.2016.04.059)
Supplement: Supplementary file 1 — Supplementary material [file mmc1.doc]

**ALL CHOLESTEROL-LOWERING INTERVENTIONS ARE EXPECTED TO REDUCE STROKE: DATA, CONFIRMATIONS AND INSIGHTS FROM IMPROVE-IT**

Raffaele De Caterina, MD, PhD^1^, Tanya Salvatore, MD^1^, and Roberto Marchioli, MD^2^

From the ^1^Institute of Cardiology and Center of Excellence on Aging, «G. d’Annunzio» University, Chieti, and ^2^Hematology & Oncology, Therapeutic Science & Strategy Unit, Quintiles, Milan, Italy

Correspondence to:

Raffaele De Caterina, MD, PhD, Institute of Cardiology – “G. d’Annunzio” University – Chieti

C/o Ospedale SS. Annunziata – Via dei Vestini – 66013 Chieti, Italy

Tel: +39-0871-41512 – FAX: +39-0871-553-461 – E-mail: [rdecater@unich.it](mailto:rdecater@unich.it)

**ALL CHOLESTEROL-LOWERING INTERVENTIONS ARE EXPECTED TO REDUCE STROKE: DATA, CONFIRMATIONS AND INSIGHTS FROM IMPROVE-IT**

Raffaele De Caterina, MD, PhD^1^, Tanya Salvatore, MD^1^, and Roberto Marchioli, MD^2^

Data in Brief – Conflict of Interest form

On behalf of my coauthors I declare NO conflict of interest as to the content of this article.


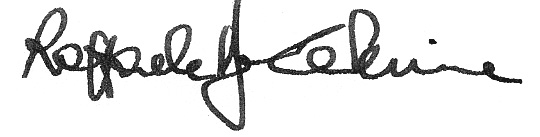


Raffaele De Caterina, MD, PhD
